# Alpha phase modulates the effectiveness and directionality of cortical communication

**DOI:** 10.1186/1471-2202-16-S1-P260

**Published:** 2015-12-18

**Authors:** Silvan C Quax, Paul Tiesinga

**Affiliations:** 1 Neuroinformatics department, Radboud University, Nijmegen, the Netherlands

## 

The selective routing of information between cortical areas is important for efficient communication in the brain. Recent experiments have shown an increase of alpha band coherence with selective attention between the pulvinar, V4, and TEO [1]. Strong cross-frequency coupling between oscillations in the alpha and gamma band [2] supports the idea that the alpha rhythm coordinates communication between higher frequency oscillations. More evidence has shown that alpha phase adjustments occur during an attentional distractor task [3]. It remains unknown however, how the alpha phase could influence selective communication. Here we investigated whether shifting the relative alpha phase between two cortical areas could coordinate cortical communication.

A network model was constructed comprised of two neuronal populations, each representing a cortical area, with each neuron modeled using the Izhikevich model [4]. Parameters were chosen such that stable gamma oscillations emerged, whereas the alpha rhythm was implemented as a periodic modulation of the input to both areas. We modulated the amplitude and relative phase of the alpha rhythm in order to investigate their effects on cortical communication, which was quantified as the coherence in the gamma frequency band.

**Figure 1:**
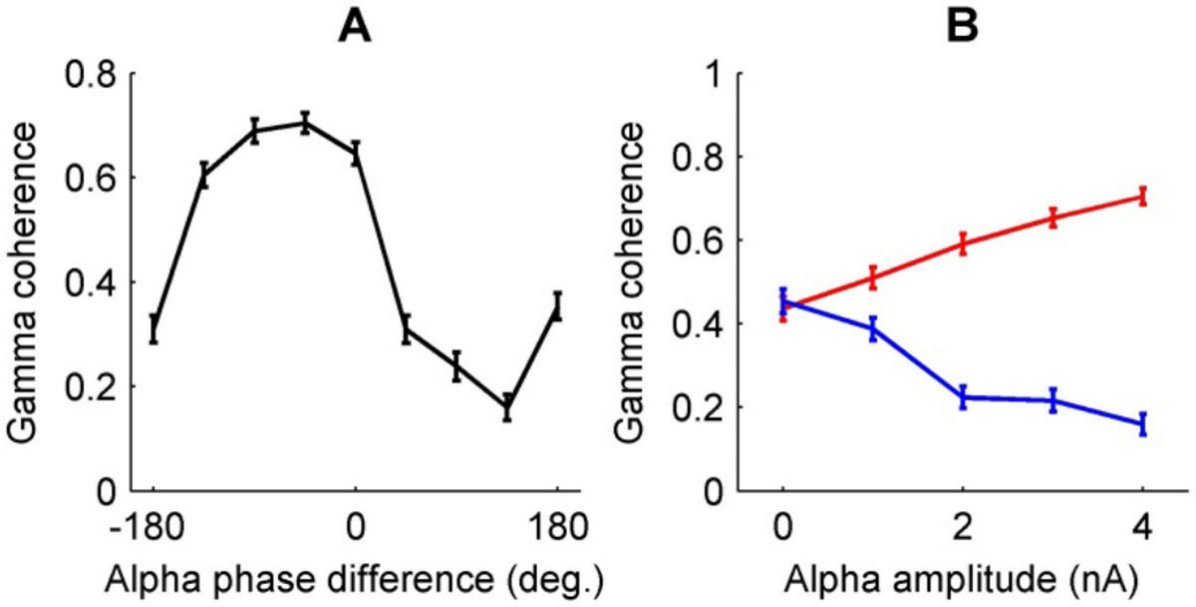
**Alpha phase influences gamma coherence**. **(A) **Coherence in the gamma band between the two neuronal populations is strongly modulated by the relative alpha phase between the two populations. **(B) **The difference in gamma coherence between best alpha phase (-45°, red line) and worst alpha phase (135°, blue line), increases with alpha amplitude.

Results showed that the relative phase of the alpha modulation between the two neuronal populations strongly affected their gamma coherence (Figure 1A). The modulation depth of the gamma coherence increased with higher alpha amplitude (Figure 1B). The relative alpha phase also had effect in a model with recurrent connectivity between the neuronal populations, where it determined the directionality of communication between the populations. Finally, the alpha phase of a neuronal population modulated its response to an external synaptic input representing a stimulus. When the relative alpha phase between the neuronal populations was optimal, a stimulus had a bigger impact on the second cortical area.

These results indicate that the relative alpha phase between neuronal populations strongly influences the effectiveness and directionality of their communication. This suggests that, during selective attention, the brain could be actively manipulating the relative phase of the alpha modulation between different cortical areas in order to coordinate the effectiveness of communication and the balance between feedforward and feedback communication between cortical areas. A prime candidate for coordinating phase shifts in the alpha rhythm is the pulvinar [1].

Taken together, our results show that the brain could coordinate cortical communication by dynamically changing the relative alpha phase between cortical areas. However, experimental manipulation of the relative alpha phase is necessary to validate this conclusion and to clarify the role of the pulvinar in this mechanism.
